# Direct comparisons of commercial weight-loss programs on weight, waist circumference, and blood pressure: a systematic review

**DOI:** 10.1186/s12889-016-3112-z

**Published:** 2016-06-01

**Authors:** Rachit M. Vakil, Ruchi S. Doshi, Ambereen K. Mehta, Zoobia W. Chaudhry, David K. Jacobs, Clare J. Lee, Sara N. Bleich, Jeanne M. Clark, Kimberly A. Gudzune

**Affiliations:** The Johns Hopkins Bloomberg School of Public Health, Baltimore, MD USA; Rutgers Robert Wood Johnson Medical School, Piscataway, NJ USA; The Johns Hopkins University School of Medicine, Baltimore, MD USA; The Johns Hopkins Bayview Medical Center, Baltimore, MD USA; The University of Maryland School of Medicine, Baltimore, MD USA; The Welch Center for Prevention, Epidemiology, Prevention, and Clinical Research, Baltimore, MD USA

**Keywords:** Commercial weight-loss programs, Weight loss, Waist circumference, Blood pressure

## Abstract

**Background:**

Obesity is common in the U.S. and many individuals turn to commercial programs to lose weight. Our objective was to directly compare weight loss, waist circumference, and systolic and diastolic blood pressure (SBP, DBP) outcomes between commercially available weight-loss programs.

**Methods:**

We conducted a systematic review by searching MEDLINE and the Cochrane Database of Systematic Reviews from inception to November 2014 and by using references identified by commercial programs. We included randomized, controlled trials (RCTs) of at least 12 weeks duration that reported comparisons with other commercial weight-loss programs. Two reviewers extracted information on mean change in weight, waist circumference, SBP and DBP and assessed risk of bias.

**Results:**

We included seven articles representing three RCTs. Curves participants lost 1.8 kg (95%CI: 0.1, 3.5 kg) more than Weight Watchers in one comparison. There was no statistically significant difference in waist circumference change among the included programs. The mean reduction in SBP for SlimFast participants was 4.5 mmHg (95%CI: 0.4, 8.6 mmHg) more than that of Atkins participants in one comparison. There was no significant difference in mean DBP changes among programs.

**Conclusions:**

There is limited evidence that any one of the commercial weight-loss programs has superior results for mean weight change, mean waist circumference change, or mean blood pressure change.

**Electronic supplementary material:**

The online version of this article (doi:10.1186/s12889-016-3112-z) contains supplementary material, which is available to authorized users.

## Background

The age-adjusted prevalence of obesity in adults in the United States is estimated to be 35 % [1]. The general prevalence of obesity has not decreased over the last 10 years, and it has increased among some groups. For example, obesity has increased by approximately 4 % among individuals aged 60 years and older [1]. Obesity has been associated with elevated blood pressure, dyslipidemia, elevated glucose level, and insulin resistance [2]. Wildman and colleagues found that 68 % of obese adults in the U.S. have two or more of these cardiometabolic abnormalities [3]. Clinically, even moderate weight loss of 5–10 % of body weight has been shown to significantly improve cardiovascular and endocrine disease risk factors [4, 5].

Among overweight and obese individuals, comprehensive lifestyle intervention alone or with adjunctive therapies is typically recommended as the initial management strategy [6]. Many individuals choose to participate in various commercially available weight-loss programs. In the U.S., these commercial programs constitute a $2.5 billion dollar industry, with Weight Watchers (45 %), Nutrisystem (14 %) and Jenny Craig (13 %) representing the largest market share [7]. A recent systematic review examined the efficacy of commercial weight-loss programs and found that individuals participating in Weight Watchers and Jenny Craig achieved significantly greater weight losses as compared to participants randomized to control/education or behavioral counseling interventions at 12 months [8]. Other programs like Nutrisystem, Medifast, and the Biggest Loser Club were also included in this review, but did not have any long-term studies of their programs. Guidelines from the American Heart Association/American College of Cardiology/The Obesity Society (AHA/ACC/TOS) state that clinicians can consider referring patients to commercial programs with demonstrated efficacy [6].

A limitation of previous reviews is that head-to-head comparisons between commercial weight-loss programs were not reported [8, 9]. Only head-to-head trials are appropriate to compare effectiveness between programs. Because participants are recruited and randomized using the same criteria, this study design reduces the potential influence of confounders that might contribute to differences in effect. Understanding whether one commercial program performs better than another is an important clinical question, as patients and clinicians are often faced with the dilemma of choosing between these weight-loss programs. Therefore, our objective in this study was to directly compare weight loss outcomes between commercially available weight-loss programs and compare changes in waist circumference and blood pressure between programs.

## Methods

### Identification and selection of weight-loss programs

We generated a list of 141 commercial and proprietary weight-loss programs from several sources, which has been described previously [8]. We included programs that encouraged dietary and behavioral change with or without physical activity that were currently available across the U.S., which resulted in a final list of 32 commercial weight-loss programs. The Johns Hopkins School of Medicine Institutional Review Board declared this study as non-human subjects research.

### Protocol, data sources and search strategy

Prior to commencing the review, we developed a study protocol, which was registered and made publically available with PROSPERO (CRD42014007155). We have previously described our methods in detail [8] in an article comparing our primary outcome of mean percentage weight change between commercial programs with control/education or behavioral counseling. We a priori established head-to-head comparisons of commercial weight-loss programs as secondary outcomes. We provide a PRISMA checklist in Additional file 1: Table S1.

We used 3 data sources to identify studies of interest: MEDLINE, the Cochrane Database of Systematic Reviews (CDSR), and the commercial weight-loss companies, which has been described previously [8]. In brief, both the MEDLINE and CDSR searches covered from database inception to November 2014 (we updated a prior MEDLINE search that covered database inception to October 2003 using a 1-year overlap to our end date) [9, 10]. Our search strategies are listed in Additional file 1: Table S2. We reviewed the reference list of each included article and relevant review articles to identify additional citations for screening. We contacted all 32 included weight-loss programs to request bibliographies of published studies and unpublished trial results, as well as reviewed each company’s website for citations.

### Study selection

Two study team members independently reviewed and screened articles using a priori eligibility criteria (Additional file 1: Table S3). All trials included overweight/obese participants. For this paper, we included RCTs of at least 12 weeks duration that directly compared weight change between 2 of the 32 commercial weight-loss programs.

### Data extraction and risk-of-bias assessment

Two team members serially extracted information regarding the study design, setting, population, intervention characteristics, mean weight change, and secondary outcomes (waist circumference, systolic blood pressure (SBP), diastolic blood pressure (DBP)) from each included study. Two reviewers independently assessed the risk of bias for each included study using the Cochrane Collaboration tool [11]. We classified a study as having “low” risk of bias if ratings were low for selection bias based on inadequate generation of a randomized sequence, detection bias based on lack of outcome assessor blinding, and attrition bias. A study had “high” risk of bias if ratings were high for any domain, “unclear” risk of bias if all domains were unclear, and “moderate” otherwise. For each program, we rated the risk of bias across trials as “low” if most studies were low; as “high” if most trials were high; and as “moderate” if otherwise.

### Data synthesis and analysis

We reported the qualitative synthesis of data for all head-to-head comparisons by calculating and displaying the between-group mean differences with 95 % CIs for each RCT, grouped by program type. We did not attempt meta-analyses given the small number of trials for each comparison.

## Results

We examined 4,212 citations, and identified seven articles [12–18] representing three RCTs reporting head-to-head comparisons that met our eligibility criteria (Additional file 1: Figure S1). Table 1 displays the population characteristics and risk of bias of each trial. Across all trials, participants’ mean age ranged from 39 to 49 years and most participants were women. Race was reported in only one trial [13], in which over three-fourths were Caucasian. All trials were rated as high risk of bias.

**Table 1:** Study and population characteristics and risk of bias of each included randomized controlled trial by program and comparator

Trial	Location population	Risk of bias^a^ -- WT	Descriptions of eligible intervention arms	Baseline characteristics				
				N	Age	Female	Race	Weight (kg)
Truby, 2006 [14] Morgan, 2008 [15]	Europe GEN	S: low D: unclear A: high Overall: high	Weight Watchers -Participants attended group classes and were reimbursed program fees. Atkins -Received copy of *Dr. Atkins New Diet Revolution* SlimFast -Participants reimbursed cost of up to two each day and provided Slim-Fast support pack	Arm 1: 58 Arm 2: 57 Arm 3: 59	Arm 1: Mean: 40 (11) Arm 2: Mean: 41 (10) Arm 3: Mean: 39 (11)	Arm 1: 42 (72 %) Arm 2: 42 (74 %) Arm 3: 42 (71 %)	NR	Arm 1: Mean: 88.8 (13.3) Arm 2: Mean: 90.3 (12.7) Arm 3: Mean: 90.1 (14.1)
Dansinger, 2005 [13]	North America GEN	S: low D: low A: high Overall: high	Atkins -4 in-person group classes during first 2 months; MVI daily -Less than 20 g CHO daily with gradual increase to 50 g daily -Received copy of *Dr. Atkins New Diet Cookbook* -60 min of exercise weekly Weight Watchers -4 in-person group classes during first 2 months; MVI daily -Track and keep points in daily goal range (~24-32 points daily) -Received copy of *Weight Watchers New Complete Cookbook* -60 min of exercise weekly	Arm 1: 40 Arm 2: 40	Arm 1: Mean: 47 (12) Arm 2: Mean: 49 (10)	Arm 1: 21 (53 %) Arm 2: 23 (58 %)	Arm 1: White: 32 (80 %) Arm 2: White: 30 (75 %)	Arm 1: Mean: 100 (14) Arm 2: Mean: 97 (14)
Lockard, 2013 [16] Galvan, 2013 [17] Simbo, 2013 [18] Dalton, 2013 [19]	North America GEN	S: high D: high A: unclear Overall: high	Curves Complete -High protein diet of 45:30, consuming 1,200 kcal/day for 1-week and 1,500 kcal/d for 11 week -30-min resistance based circuit interspersed with callisthenic exercises or Zumba 4 days per week -Access to online individualized weekly meal plans, daily motivational and educational videos, and weekly personal coaching sessions Weight Watchers -Followed the Weight Watchers® Points Plus program -Followed food plans based on a points system -Attended weekly meetings for weigh-ins and presentations regarding exercise recommendations, tracking methods, and weight reduction strategies -Exercise encouraged but not mandatory Jenny Craig -Received meals delivered to their home for 12 weeks -Participated in a weekly phone consultation to discuss weight changes, exercise encouragement, and online tracking methods -Exercise encouraged but not mandatory Nutrisystem -Received meals delivered to their home for 12 weeks -Participated in a weekly phone consultation to discuss weight changes, exercise encouragement, and online tracking methods -Exercise encouraged but not mandatory	Arm 1: 23 Arm 2: 29 Arm 3: 27 Arm 4: 28	Overall: Mean: 46 (12)	Overall: 100 %	NR	NR

^a^Risk of bias assessed using three questions from the Cochrane Collaboration’s tool [11]: selection bias selection bias due to inadequate generation of a randomized sequence, detection bias due to lack of outcome assessor blinding, and attrition bias. Attrition bias rated as low if analysis type was intention-to-treat with baseline observation carried forward or equivalent, attrition was <30 %, and difference in attrition between arms was <20 %; rated as moderate if analysis type was intention-to-treat with last observation carried forward or otherwise as indicated in table, attrition was <30 %, and difference in attrition between arms was <20 %; rated as a high if analysis type was completers’ only or attrition ≥30 % or difference in attrition between arms ≥20 %. We designated a trial’s overall risk of bias as low at a time point, if selection bias, detection bias, and attrition bias were all low. If all domains were unclear, we labeled the trial as unclear. We designated the trial as high, if at least one of the above domains were rated as high. Risk of bias was otherwise moderate. *Abbreviations*: *A* attrition bias due to amount, nature, or handling of incomplete outcome data, *CHO* carbohydrates, *D* detection bias due to knowledge of the allocated interventions by outcome assessment (lack of outcome assessor blinding), *GEN* study population consisted of patients with overweight and obesity, *JC* Jenny Craig, *MR* meal replacements, *NR* not reported, *S* selection bias due to inadequate generation of a randomized sequence, *WT* weight outcome

**Fig. 1:**
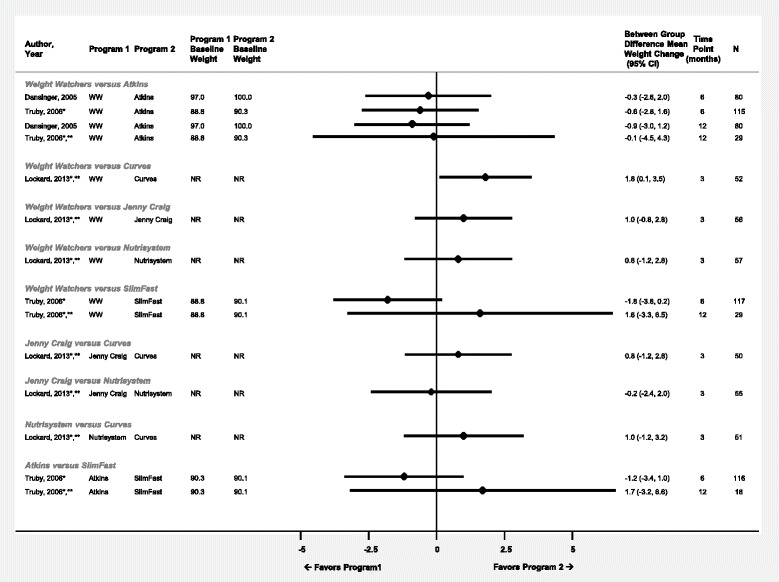
Difference in mean weight change (kg) between commercial programs displayed by comparison and time point. Diamond size is standardized across trials and does not reflect sample size analyzed. *Results reported in more than one article or data source. **Results from completers’ analysis or unclear analysis type. Abbreviations: NR – not reported; WW – Weight Watchers

**Fig. 2:**
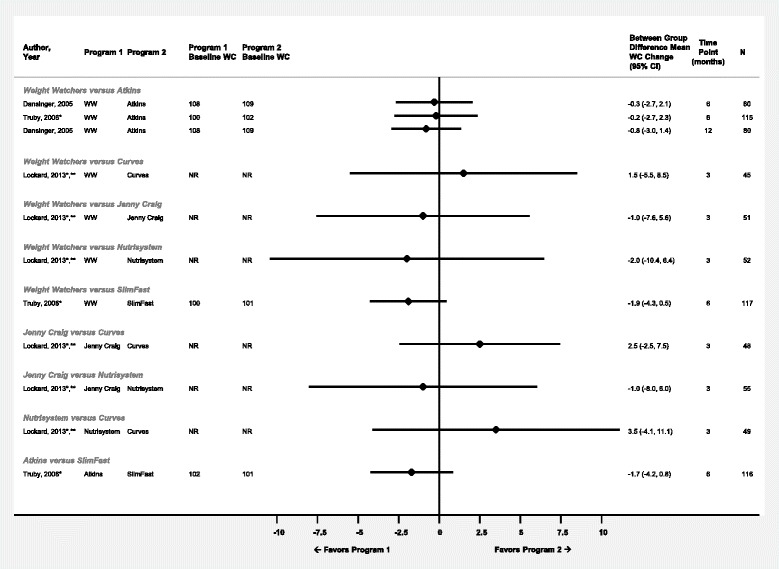
Difference in mean waist circumference change (cm) between commercial programs displayed by comparison and time point. Diamond size is standardized across trials and does not reflect sample size analyzed. *Results reported in more than one article or data source. **Results from completers’ analysis or unclear analysis type. Abbreviations: NR – not reported; WC – waist circumference; WW – Weight Watchers

**Fig. 3:**
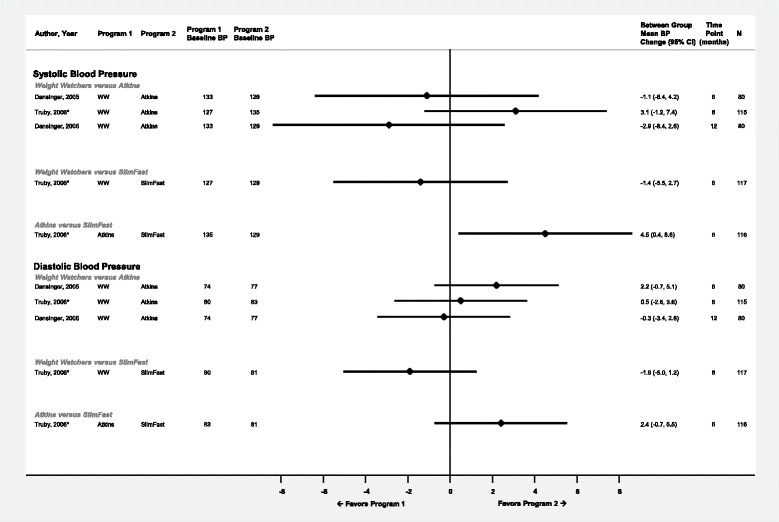
Differences in mean systolic and diastolic blood pressure changes (mmHg) between commercial programs displayed by comparison and time point. Diamond size is standardized across trials and does not reflect sample size analyzed. *Results reported in more than one article or data source. **Results from completers’ analysis or unclear analysis type. Abbreviations: BP – blood pressure; NR – not reported; WW – Weight Watchers

Three studies reported multiple head-to-head comparisons (Weight Watchers vs. Atkins [12–14], Curves [15–18], Jenny Craig [15–18], Nutrisystem [15–18], and SlimFast [13, 14]; Jenny Craig vs. Curves [15–18], Nutrisystem [15–18]; Nutrisystem vs. Curves [15–18]; and Atkins vs. SlimFast [13, 14]). Of the fourteen head-to-head comparisons of mean weight change available, only one showed a significant difference (Fig. 1) where Curves participants lost 1.8 kg (95%CI: 0.1, 3.5 kg) more than Weight Watchers participants at three months [15]. There was no significant difference between programs for mean waist circumference change at any time point, among the eleven available comparisons (Fig. 2). Two trials reported data for the difference in SBP and DBP changes between programs (Fig. 3) [12, 13]. A comparison of Atkins versus SlimFast showed that SlimFast participants had an average of 4.5 mmHg (95 %CI: 0.4, 8.6 mmHg) lower systolic blood pressure after six months of follow-up [13]. There was no significant difference in diastolic blood pressure change among the reported data.

## Discussion

While previous literature has shown that Weight Watchers, Jenny Craig and Nutrisystem are effective in facilitating weight loss as compared to control/education or behavioral counseling [8], clinicians and patients may want to directly compare available commercial weight-loss programs when discussing the options for losing weight. In the present study, we found that most commercial programs perform similarly with respect to short-term changes in weight, waist circumference, and blood pressure among eligible head-to-head RCTs included in this review. Our findings build upon a recent review that compared named diets like Ornish and Atkins, and found that weight loss differences between individual strategies were small [19]. We considered commercial programs such as SlimFast and Curves not included in the review by Johnston and colleagues [19], and examined other outcomes beyond weight.

Previous surveys have found that 10 to 15 % of patients are using commercial programs to lose weight [20, 21]. Participating in these programs has been associated with greater likelihood of achieving a clinically significant weight loss of 10 % or greater [22]. While the AHA/ACC/TOS guidelines recommend that clinicians refer obese patients to high-intensity, comprehensive lifestyle interventions that include a moderately reduced calorie diet, increased physical activity, and use of behavioral strategies to enhance adherence [6], a recent study found that few programs in the community meet these recommendations [23]. In contrast, commercial weight-loss programs are widely available across the U.S. and some programs’ practices are consistent with the AHA/ACC/TOS suggested elements. Four programs included in this review (Weight Watchers, Curves, Jenny Craig, Nutrisystem) have practices consistent with these elements. In fact, the AHA/ACC/TOS guidelines suggest that commercial programs with demonstrated efficacy may be acceptable [6]. While counseling programs that promote healthy diet and physical activity have a role in treating obesity, evidence also suggests that a role exists for commercial programs in weight management.

While this review reported multiple head-to-head comparisons, we identified only three studies that met our inclusion criteria – one of which was an unpublished trial. Additional head-to-head RCTs would benefit clinicians and patients, as these data would provide clear information to determine whether one program is superior or similar to another. Health insurers might be considering offering a commercial weight-loss program as a benefit [24]. Direct comparisons of these programs would also help better inform policymakers’ selection of a program. Given the costs of these programs, patients and insurers may not be able to afford more than one option, thus rigorous comparative effectiveness research is needed to inform these decisions. Trials that directly compare commercial weight-loss programs head-to-head are the most appropriate methodology to compare effectiveness. Individuals may be tempted to use the recent review reporting comparisons of commercial weight loss programs to control/education or counseling to compare programs; however, they might draw inappropriate conclusions. For example, as compared to control at 3 months, Weight Watchers achieved 2.5–5.9 % greater weight loss whereas Nutrisystem had a 6.7 % greater loss [8]. Some may subsequently interpret this finding as Nutrisystem resulting in greater weight loss than Weight Watchers. However, we found that the direct comparison of these two programs were not significantly different at 3 months. While we would not make definitive conclusions based on one trial, the finding does illustrate the importance of direct comparisons to understand whether one commercial program is superior to another and the need for more head-to-head trials. In addition, such trials should extend to 12 months or beyond to provide a comparison of programs’ long-term effectiveness.

The limited information available for blood pressure outcomes is also critical, as some programs used prepackaged, processed foods that may contain higher levels of sodium than recommended [25]. Future studies should include lipids, glucose and hemoglobin A1c as outcomes, as dyslipidemia and diabetes mellitus are conditions where weight loss is often indicated.

Our study has several strengths. We considered a comprehensive list of commercial weight-loss programs when searching the evidence, and focused only on RCTs to capture evidence that has the greatest scientific rigor. Some commercial weight-loss programs may only conduct prospective or retrospective case series studies to describe their effect, which are subject to biases including selection bias. While limiting our synthesis to only RCTs increases the scientific rigor, some comparative studies may have been excluded. We also considered other outcomes beyond weight loss, which have not been considered in other reviews [8, 9, 19]. The present study also has several limitations. We used two databases to conduct our searches and obtained references from the commercial programs themselves; however, we might have missed citations listed only in other databases such as EMBASE or Web of Science. We included only RCTs of commercial weight-loss programs available throughout the United States. The evidence base also has several limitations. The risk of bias was high for all three of the included studies. Most trials reported only short-term results at three months; therefore, we cannot comment on between-program differences in maintenance of outcomes.

## Conclusion

Overall, our results provide preliminary evidence of little differences in short-term benefits with respect to mean weight change, mean waist circumference change, and mean blood pressure change between commercial weight-loss programs in head-to-head comparisons. Additional long-term head-to-head trials of commercial programs would help clinicians who may be considering referring patients to these programs to achieve and maintain clinically significant weight loss.

## Abbreviations

AHA/ACC/TOS, American Heart Association/American College of Cardiology/The Obesity Society; CDSR, cochrane database of systematic reviews; DBP, diastolic blood pressure; RCT, randomized controlled trial; SBP, systolic blood pressure

## Additional file

Additional file 1:
**Table S1.** PRISMA Checklist. **Table S2.** Electronic Search Strategy. **Table S3.** Study Eligibility Criteria. **Figure S1.** Summary of evidence search and selection. *Other exclusions included trials with ineligible study designs (retrospective case series, RCT < 12 weeks duration, etc.) or ineligible programs (not available in the US, etc.). **Ineligible commercial programs include those that use medications or supplements, modified specifically for the study, unavailable in the U.S., or available only to special populations like active duty military or veteran. Abbreviations: CDSR – Cochrane Database of Systematic Reviews; RCT – randomized controlled trial. (DOC 1403 kb)
