# Supplementary material for: Direct comparisons of commercial weight-loss programs on weight, waist circumference, and blood pressure: a systematic review
Source: BMC Public Health. 2016 Jun 1;16:460. doi: 10.1186/s12889-016-3112-z (PMC4888663; doi:10.1186/s12889-016-3112-z)
Supplement: Additional file 1: — Table S1. PRISMA Checklist. Table S2. Electronic Search Strategy. Table S3. Study Eligibility Criteria. Figure S1. Summary of evidence search and selection. *Other exclusions included trials with ineligible study designs (retrospective case series, RCT < 12 weeks duration, etc.) or ineligible programs (not available in the US, etc.). **Ineligible commercial programs include those that use medications or supplements, modified specifically for the study, unavailable in the U.S., or available only to special populations like active duty military or veteran. Abbreviations: CDSR – Cochrane Database of Systematic Reviews; RCT – randomized controlled trial. (DOC 1403 kb) [file 12889_2016_3112_MOESM1_ESM.doc]

**Additional files: Tables & Figures**

Table S1. PRISMA Checklist

Table S2. Search Strategies Used, by Database

Table S3. Study Eligibility Criteria

Figure S1. Summary of Evidence Search and Selection


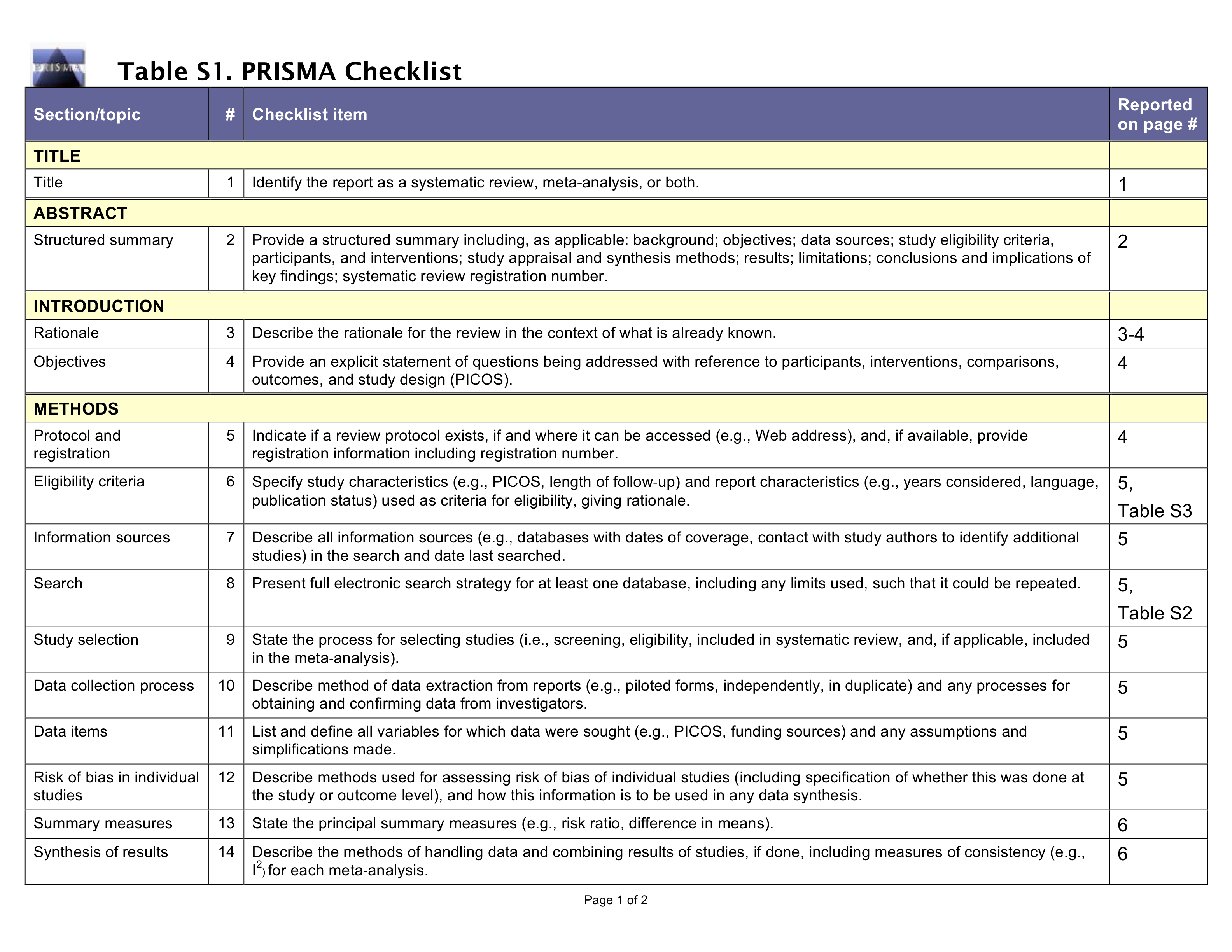


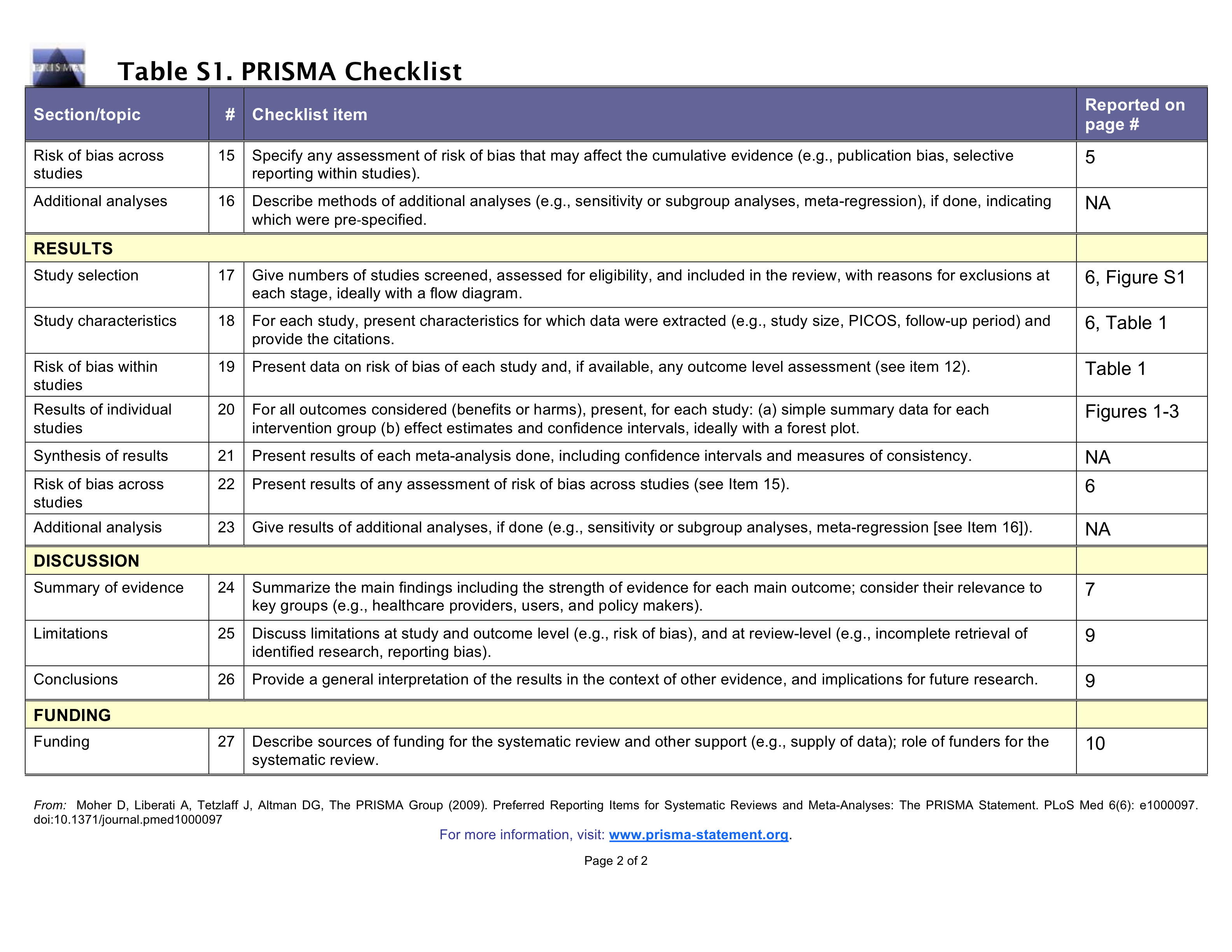


| **Table S2. Search Strategies Used, by Database** | | |
| --- | --- | --- |
| **Database** | **Search Dates** | **Search Strategy** |
| MEDLINE | 10/2002–11/2014 | Subject headings obesity and weight loss combined with each of the following terms: weight reduction programs, diet reducing, nutrition, behavior therapy, cognitive therapy, exercise, internet, self-help groups |
| MEDLINE | 10/2002–11/2014 | Keyword search for the terms: weight loss and commercial, weight loss and proprietary |
| MEDLINE | Inception–11/2014 | Keyword search for each included commercial program combined with the terms: weight loss and commercial or proprietary |
| CDSR | Inception–11/2014 | MeSH and keyword search for obesity and weight loss combined with each of the following MeSH and key word terms: weight reduction programs, diet reducing, nutrition therapy, behavior therapy, cognitive therapy, exercise, internet, self-help groups |
| CDSR | Inception–11/2014 | MeSH and keyword search for: obesity and weight loss and commerce, obesity and weight loss and proprietary health facilities |
| *Abbreviations: CDSR – Cochrane Database of Systematic Reviews; MeSH – Medical Subject Headings.* | | |

| **Table S3. Study Eligibility Criteria** | |
| --- | --- |
| **Population and condition of interest** | Adults who are overweight or obese (BMI ≥25 kg/m^2^). The following exclusions were made as such populations are not representative of the U.S. general public aiming to lose weight:  *Excluded* studies if they included only children  *Excluded* studies of underweight or normal weight adults (BMI<25 kg/m^2^)  *Excluded* studies of cancer survivors |
| **Interventions and approaches** | Studies must have evaluated a commercial or proprietary weight loss program, which we identified by the explicit naming of the program or referencing the proprietary literature or commercial product as their intervention. The following exclusions were made as such programs are not representative of those offered to and/or are inappropriate for the U.S. general public aiming to lose weight:  *Excluded* commercial or proprietary programs tailored for an individual center, unique population (e.g., military or veterans) or employer-based versions of programs (e.g., commercial program tailored to workplace setting with unique environmental elements not available to the comparator group)  *Excluded* programs focused on food addiction  *Excluded* residential weight loss programs  *Excluded* any program that evaluates or uses medications and/or non-FDA-approved supplements that included dietary supplements, nutraceuticals or other products (e.g., vitamins, minerals, herbals, botanicals, amino acids, enzymes, hormones – meal replacements are not included in our definition of supplements) as these products are not appropriate for all patients in a general population and supplements have variable efficacy and safety  *Excluded* programs without weight loss as the primary goal (e.g., wellness, cardiovascular risk reduction)  *Excluded* commercial or proprietary programs not available in the U.S. or available only in a single state |
| **Comparisons of interest** | Included comparisons with other commercial or proprietary weight loss programs (as defined above)  *Excluded* studies with control or education comparator only  *Excluded* studies with behavioral counseling comparator |
| **Outcomes and Timing** | Weight outcomes including mean weight change, mean waist circumference change, and mean blood systolic and diastolic blood pressure changes at 12 weeks and beyond  *Excluded* outcomes reported prior to 12-week time point |
| **Type of study** | Included studies from the prior report that met all other criteria Included randomized controlled trials ≥12 weeks duration comparing an eligible intervention to eligible comparator  *Excluded* qualitative studies  *Excluded* studies with no original data (e.g., reviews, editorials, comments, letters)  *Excluded* randomized controlled trials examining an eligible intervention without an eligible comparator  *Excluded* randomized controlled trials <12 weeks duration or unspecified duration  *Excluded* prospective case series and retrospective case series  *Excluded* studies with other observational designs *Excluded* studies published only as abstracts  *Excluded* non-English publications |
| *Abbreviations: BMI – body mass index; FDA – Food and Drug Administration* | |


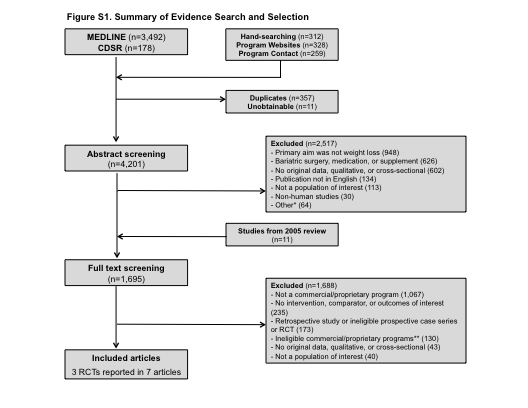


**Figure S1. Summary of evidence search and selection.** *Other exclusions included trials with ineligible study designs (retrospective case series, RCT<12 weeks duration, etc.) or ineligible programs (not available in the US, etc.). **Ineligible commercial programs include those that use medications or supplements, modified specifically for the study, unavailable in the U.S., or available only to special populations like active duty military or veteran. Abbreviations: CDSR – Cochrane Database of Systematic Reviews; RCT – randomized controlled trial.
